# Validation data for the use of bradykinin and substance P protease activity assays with capillary blood and blood cards

**DOI:** 10.1016/j.dib.2019.104873

**Published:** 2019-11-23

**Authors:** Ulrich Schreiber, Malte Bayer, Simone König

**Affiliations:** Core Unit Proteomics, Interdisciplinary Center for Clinical Research, Medical Faculty, University of Münster, Germany

**Keywords:** Bradykinin, Substance P, Thin-layer chromatography, Dried blood spots, Protease activity, Angiotensin-converting enzyme

## Abstract

In the associated main paper (“Labeled substance P as a neuropeptide reporter substance for enzyme activity” (http://doi.org/10.1016/j.jpba.2019.112953)), substance P was shown to be a valuable neuropeptide reporter substance to monitor the protease activity of serum. The assay was developed based on the predecessor assay using bradykinin (“A vote for robustness: Monitoring serum enzyme activity by thin-layer chromatography of dabsylated bradykinin products”, http://doi.org/10.1016/j.jpba.2017.06.007). Both neuropeptides are of interest in inflammation and pain research and were thus explored for use with capillary blood and blood cards (see associated MethodX paper “Neuropeptide reporter assay for serum, capillary blood and blood cards”). Here, we present validation data for the assay when sampling with blood cards as well as data on the use of fresh capillary blood.

**Table:** Specifications Table

Subject	Analytical Chemistry
Specific subject area	Protease activity in capillary blood (fresh and dried on blood cards) is studied by observation of the fragments of neuropeptide reporter substances
Type of data	Table Image Chart Figure
How data were acquired	Thin-layer chromatography and image analysis Scanner: Canon 9000F Mark2 Software: Photoshop plug-in Silver Efex Pro (Google, Mountain View, USA), JustTLC (Sweday, Sodra Sandby, Sweden)
Data format	Raw and analysed data in Excel
Parameters for data collection	The protocol is available in the associated MethodX article “Neuropeptide reporter assay for serum, capillary blood and blood cards”. Parameters in brief: 1 μl of capillary blood, incubation at 37 °C for 1 h, thin-layer chromatography mobile phase CHCl_3_/methanol/H_2_O/CH3COOH, 11 : 4: 0.6 : 0.09 v/v/v/v
Description of data collection	The protocol is available in the associated MethodX article “Neuropeptide reporter assay for serum, capillary blood and blood cards”. Briefly, capillary blood was drawn and incubated with dabsylated bradykinin and substance P, respectively. Two major fragments were monitored in each case. They were separated using thin-layer chromatography (TLC). TLC-plates were scanned and the spot intensities were analysed.
Data source location	IZKF Core Unit Proteomics, University of Münster, Germany
Data accessibility	With the article
Related research article	[1] U. Schreiber*, C. Engl*, M. Bayer, S. König, Labeled substance P as a neuropeptide reporter for enzyme activity. J. Pharmaceut. Biomed. Anal. (2019) http://doi.org/10.1016/j.jpba.2019.112953

**Table:** 

**Value of the Data** • This data is useful, because it shows replicate measurements of the assay when applied to blood cards. • Researchers interested in inflammation and pain benefit from the data. • The data can be further used as base values and for comparison to their own data by other groups. • The additional value of this data is their use for the evaluation of biological variance.

## 1 Data description

This Data-in-Brief article is associated with a publication where the use of dabsylated substance P (DSP) as a neuropeptide reporter substance (NRS) for the monitoring of protease activity (PA) in serum is demonstrated (see Ref. [1] and all references therein). In an earlier work, bradykinin was used for the same purpose (http://doi.org/10.1016/j.jpba.2017.06.007). The degradation of dabsylated bradykinin (DBK) visualized the lowered protease activity in patients suffering from chronic pain. Both bradykinin and substance P are of interest in inflammation and pain research. The assay moreover requires very little sample volume (<10 μl) so that the use of blood cards was evaluated. These simplify blood collection and enable outpatient sampling.

The assay is based on the incubation of NRS with blood or serum and the detection of the generated enzymatic fragments by thin-layer chromatography (TLC). The complete protocol is available in the associated MethodX article. Analysis was based on the relative spot intensity values of the individual fragments with respect to the total spot intensities of DBK and its fragments. Blood was obtained from healthy volunteers observing the declaration of Helsinki. Validation data are presented below (for raw data see Supplement).

**Fig. 1:**
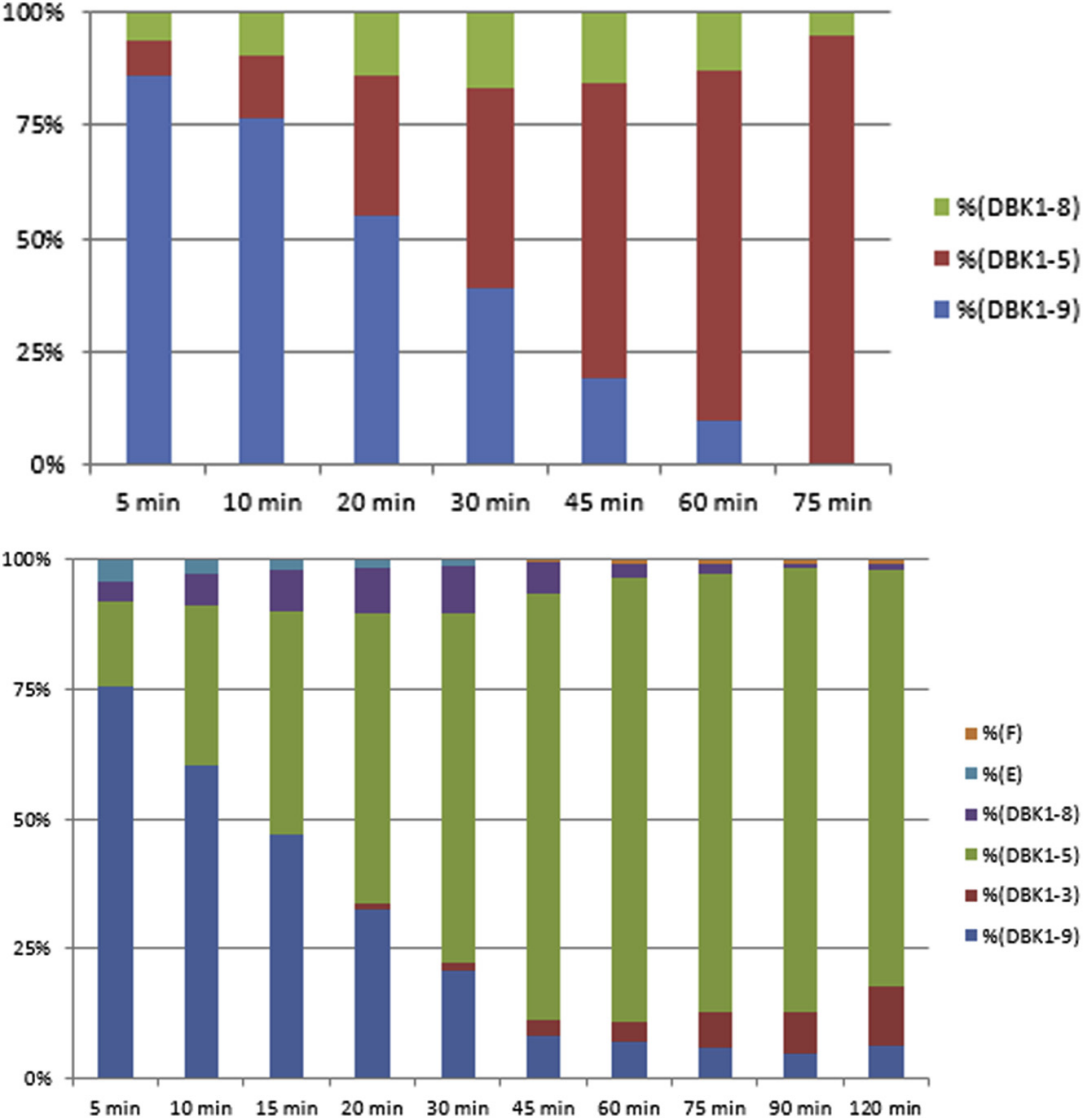
Time series for the degradation of DBK in 1 μl blood (bottom panel) or blood equivalent extracted from a dried blood spot (top, healthy male, 29 y). Relative values. Labels F and E stand for so far not identified weak components.

For DBK, the fragments DBK1-8 and DBK1-5 were monitored as described. In healthy probands, 70–90% of DBK were degraded by 1 μl equivalent of extracted dried blood in 1 h (Fig. 1). Degradation time was shorter, when more blood equivalent was used.

**Fig. 2:**
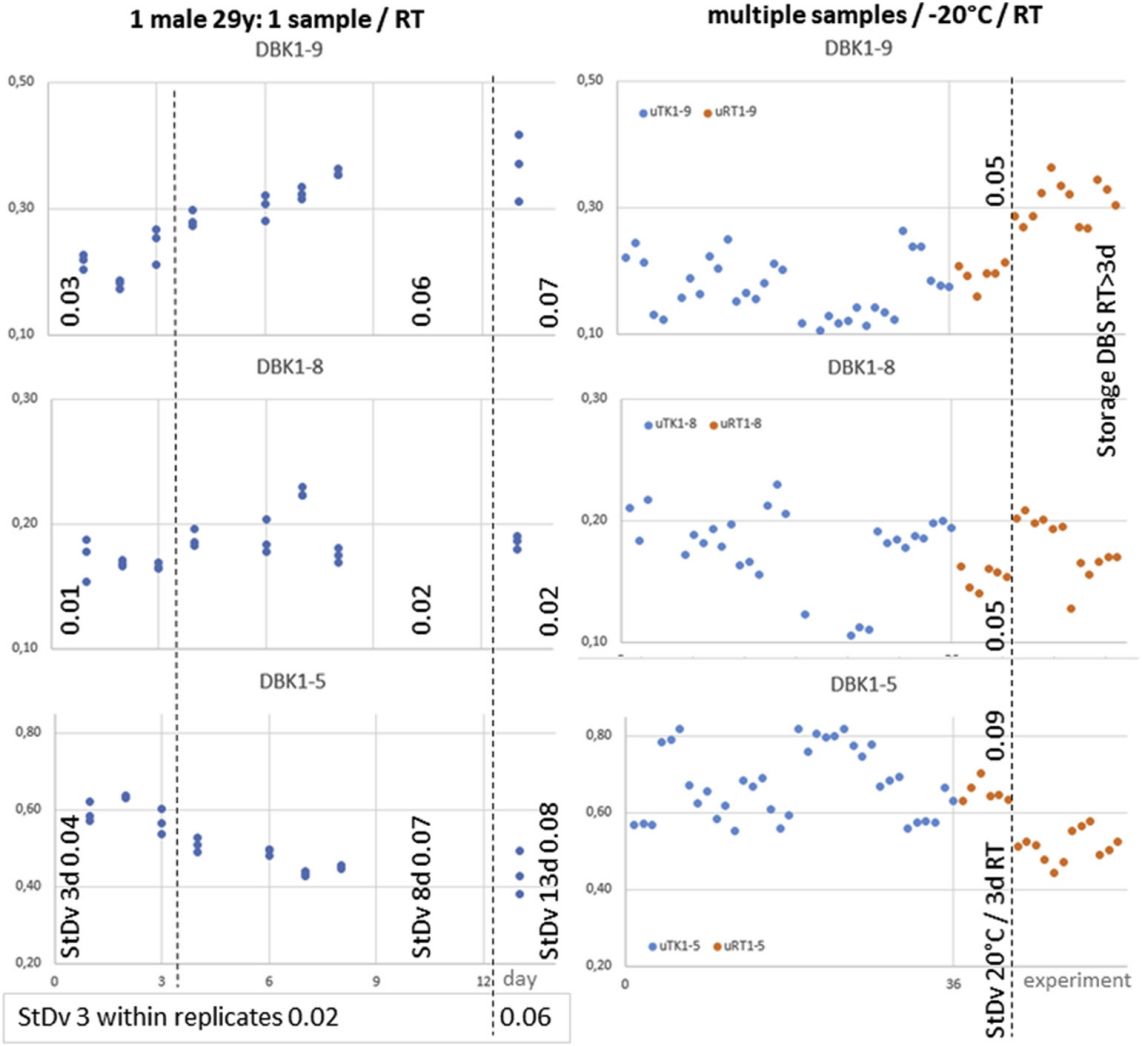
Replicate experiments with blood cards. Healthy male volunteer (29 years old). Left panel: Storage of one sample for up to 13 days. Right panel: Storage of blood cards at −20 °C or at room temperature (red). Relative Values.

**Fig. 3:**
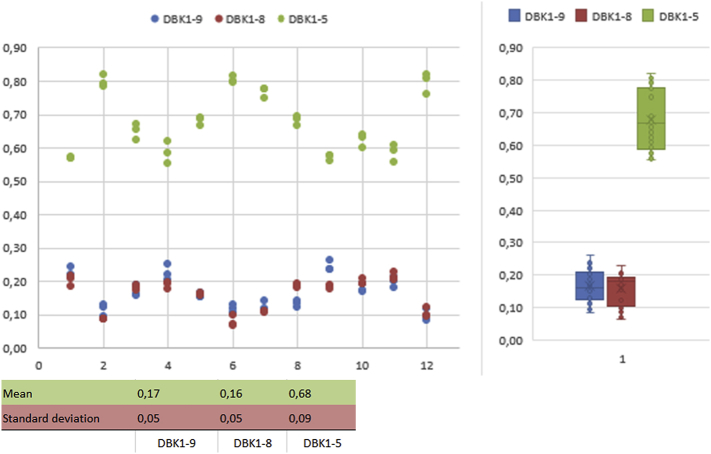
Replicate experiments (12 × 3) with blood cards. Healthy male volunteer (29 y). Experiments were performed on different days on different TLC-plates. Sampling was performed on different days. Relative values.

**Fig. 4:**
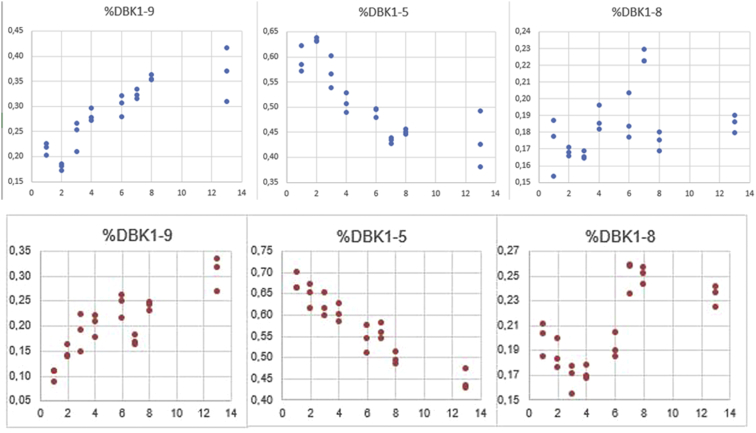
Replicate experiments with blood cards (healthy males, top: 29 y, bottom: 34 y). Relative values versus storage time in days. Standard deviations were between 0.002 und 0.044 (day 13, top) and 0.005 und 0.030 (bottom panel).

In order to exclude variation due to hormonal changes, the variation in male volunteers was studied (Fig. 2, Fig. 3, Fig. 4). Standard deviation both on a single TLC-plate and on several plates was <0.03. Variation increased with storage time of the blood card. Storage at room temperature for more than three days did not only increase the measurement error it also lowered the protease activity in dried blood. The standard deviation for 36 cooled samples of DBK1-5 of the same person was 0.09; it was even lower for the other parameters.

Fig. 3 shows experiments with three replicates each on a single TLC-plate (storage at −20 °C). Sampling was performed on different days. Between-day variation was expectedly larger than inner-day variation, technical variation was smaller than biological variation.

**Fig. 5:**
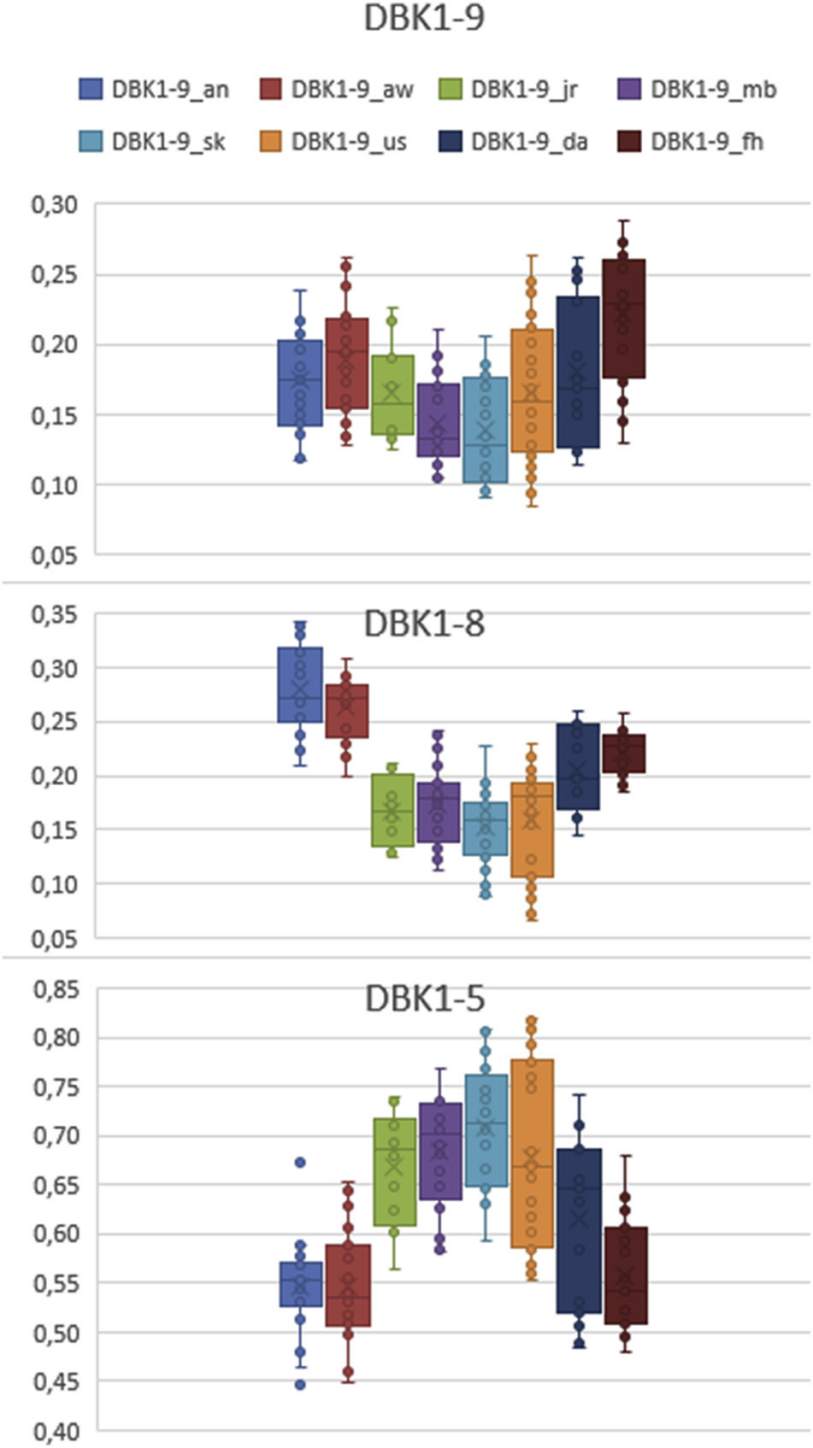
Box plots of experiments with blood cards. Eight volunteers (male and female, Table 1).

**Fig. 6:**
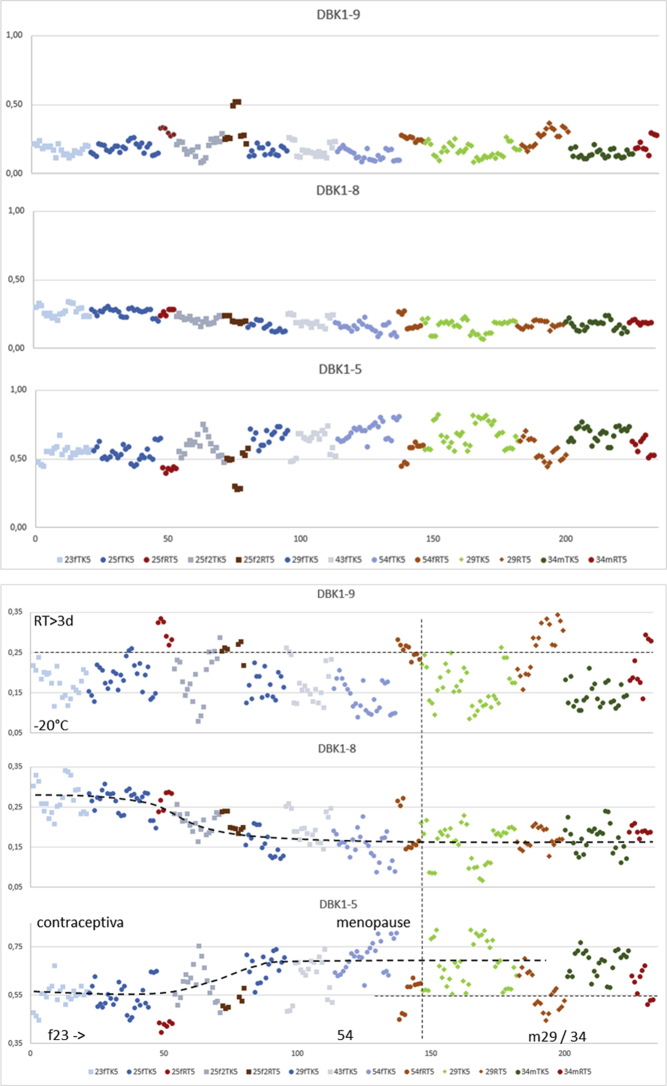
Replicate experiments with blood cards. Healthy volunteers (6 female, 2 male, bottom: zoom). Storage at room temperature in red. Age range 23–54 y (increasing from left).

In order to test biological variation, blood cards with capillary blood from both male and female volunteers were run in 1 h experiments. Three replicates each were examined on one TLC plate (Fig. 5, Fig. 6; Table 1).

**Table 1:** Measurements for blood cards of eight volunteers (23–54 y, average values and standard deviations of 12–30 discreet values).

#	Value	Age	Gender		DBk1-9	DBk1-8	DBk1-5		DBk1-9	DBk1-8	DBk1-5
an	18	23	w	ø	0,18	0,29	0,53				
								stAbw	0,04	0,04	0,05
aw	20	25	w	ø	0,18	0,26	0,58				
								stAbw	0,04	0,03	0,06
da	15	43	w	ø	0,20	0,22	0,58				
								stAbw	0,05	0,04	0,09
jr	12	29	w	ø	0,17	0,17	0,67				
								stAbw	0,03	0,03	0,06
mb	24	34	m	ø	0,14	0,18	0,68				
								stAbw	0,03	0,04	0,05
sk	24	54	w	ø	0,14	0,16	0,70				
								stAbw	0,04	0,04	0,06
us	30	29	m	ø	0,17	0,15	0,68				
								stAbw	0,05	0,05	0,09
fh	18	25	w	ø	0,25	0,23	0,52				

**Fig. 7:**
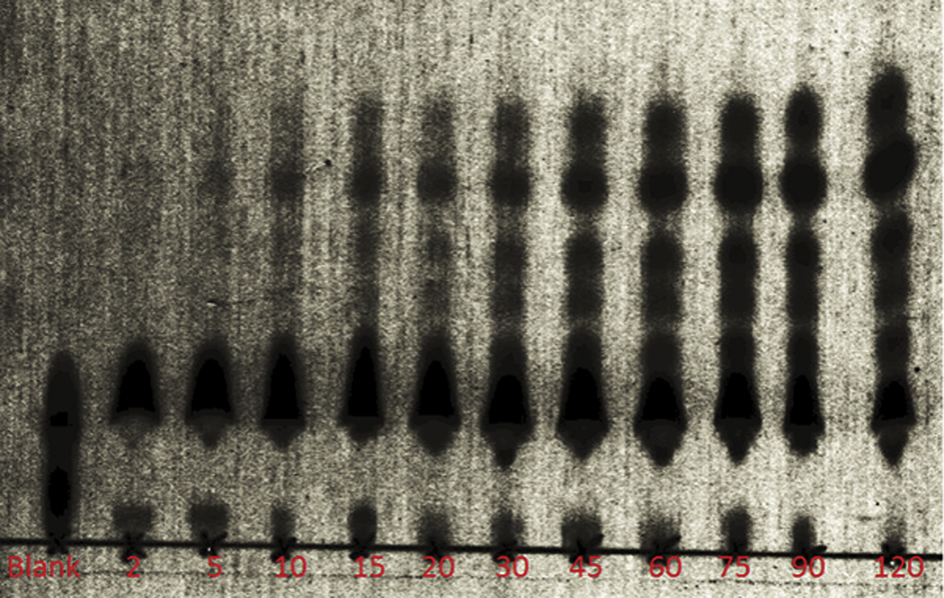
Time series with blood cards for K3DSP.

Most evaluation experiments with blood cards have been performed using DBK so far. An initial test for K3DSP is shown in Fig. 7.

## 2 Experimental design, materials, and methods

Comprehensive description of experimental protocol is available in the associated MethodX article “Neuropeptide reporter assay for serum, capillary blood and blood cards”. Briefly, capillary blood was drawn from healthy volunteers (observing the declaration of Helsinki) and incubated with dabsylated bradykinin and substance P, respectively (Peptide Specialty Laboratories, Heidelberg, Germany). Two major enzymatic fragments were monitored in each case. They were separated using thin-layer chromatography (TLC). TLC-plates were scanned and the spot intensities were analysed. One microliter of capillary blood was sufficient. The incubation proceeded at 37 °C for 1 h. The TLC mobile phase was CHCl_3_/methanol/H_2_O/CH3COOH, 11 : 4: 0.6 : 0.09 v/v/v/v. For scanning a regular office scanner was used (Canon 9000F Mark2). Images were analysed using Photoshop plug-in Silver Efex Pro (Google, Mountain View, USA) and JustTLC (Sweday, Sodra Sandby, Sweden).
